# Drug resistance and minimal residual disease in multiple myeloma

**DOI:** 10.20517/cdr.2021.116

**Published:** 2022-02-16

**Authors:** Alessandro Gozzetti, Sara Ciofini, Anna Sicuranza, Paola Pacelli, Donatella Raspadori, Emanuele Cencini, Dania Tocci, Monica Bocchia

**Affiliations:** Hematology, University of Siena, Azienda Ospedaliera Universitaria Senese, Siena 53100, Italy.

**Keywords:** Multiple myeloma, minimal residual disease, drug resistance, therapy, next-generation flow cytometry

## Abstract

Great progress has been made in improving survival in multiple myeloma (MM) patients over the last 30 years. New drugs have been introduced and complete responses are frequently seen. However, the majority of MM patients do experience a relapse at a variable time after treatment, and ultimately the disease becomes drug-resistant following therapies. Recently, minimal residual disease (MRD) detection has been introduced in clinical trials utilizing novel therapeutic agents to measure the depth of response. MRD can be considered as a surrogate for both progression-free and overall survival. In this perspective, the persistence of a residual therapy-resistant myeloma plasma cell clone can be associated with inferior survivals. The present review gives an overview of drug resistance in MM, i.e., mutation of β5 subunit of the proteasome; upregulation of pumps of efflux; heat shock protein induction for proteasome inhibitors; downregulation of *CRBN *expression; deregulation of *IRF4* expression; mutation of *CRBN*, *IKZF1*, and *IKZF3 *for immunomodulatory drugs and decreased target expression; complement protein increase; sBCMA increase; and BCMA down expression for monoclonal antibodies. Multicolor flow cytometry, or next-generation flow, and next-generation sequencing are currently the techniques available to measure MRD with sensitivity at 10^-5^. Sustained MRD negativity is related to prolonged survival, and it is evaluated in all recent clinical trials as a surrogate of drug efficacy.

## INTRODUCTION

Multiple myeloma (MM) represents the second most frequent hematological malignancy, and significant survival improvements in the years have been seen^[1-3]^. Survival has ameliorated thanks to the availability of novel classes of drugs such as proteasome inhibitors (PIs: bortezomib, and carfilzomib), immunomodulatory drugs (IMIDs: thalidomide, lenalidomide, and pomalidomide), monoclonal antibodies (moAb: daratumumab, elotuzumab, isatuximab, and belantamab mafodotin). These new drugs are utilized alone or combined as triplets or quadruplets in the treatment of relapsed/refractory patients and thereafter at diagnosis with good results also in extramedullary MM^[4-17]^. MM has important clinical heterogeneity and complex genetic abnormalities^[18]^. Cytogenetic analysis and fluorescence *in situ* hybridization can help to distinguish different categories and risk-stratify MM patients^[19]^. In particular, t(4;14), t(14;20), gain of chromosome 1q, and deletion 17p give a poorer outcome, while t(11;14) and t(14;16) seem to have an intermediate prognosis^[20-22]^. In this view, relapses are seen in the majority of patients, and some of them still have a dismal prognosis. However, while cytogenetic analysis can help to stratify prognosis, it does not fully explain initial MM drug resistance (DR). Two main factors seem to emerge: (1) acquired drug resistance; and (2) sub-clonal heterogeneity^[23,24]^. Mechanisms of acquired drug resistance to the main classes of new drugs available in MM have been extensively reviewed recently^[22]^. A novel method to detect minimal residual disease has been developed recently^[23-35]^, i.e., MRD detected by next-generation flow (NGF) or next-generation sequencing (NGS), which have been reported as important tools by the International Myeloma Working Group (IMWG) in the recent response guidelines^[36]^. MRD is an important surrogate for survival, as well as progression-free (PFS) and overall survival (OS)^[37]^. In particular, sustained MRD negativity confirmed at one year is of great importance to predict clinical outcome of MM patients. MRD can also detect sub-clones that acquire higher genomic instability and can ultimately drive resistance^[38]^. This review summarizes the main DR cell-inherent/intrinsic and extrinsic mechanisms to novel drugs in MM and will focus on recent developments regarding MRD as a tool to predict PFS and OS in clinical trials.

### Drug resistance in MM

Drug resistance is the leading cause of a relapsed/refractory disease, and it can ultimately decrease survival. Many novel drugs are now available in MM with many used at diagnosis in triplet or quadruplet. MM patients can develop DR after a few cycles of therapy in a variable manner. The main mechanisms of resistance to chemotherapeutic drugs in MM can involve a drug-efflux pump, such as P-glycoprotein, or other mechanisms that inhibit the drug to enter the cell. Moreover, enzymatic inactivation of the drug or adhesion to bone marrow stromal cells, such as fibroblasts and immune cells including macrophages, in the microenvironment can be mentioned. To develop strategies for the future treatment of DR-MM patients, the main mechanisms of resistance to the different classes of drugs, namely proteasome inhibitors, immunomodulatory drugs, and monoclonal antibodies, have to be elucidated. The principal mechanisms of resistance to novel drugs and resistance escape are reported in Table 1.

**Table 1:** Principal mechanisms of drug resistance and resistance escape

**C**	**D**	**M of A**	**DR**	**Resistance escape**	**Ref.**
moAb	Dara Isa Elo Bela	ADCC, CDC, macrophage-mediated phagocytosis, apoptosis via Fc-mediated crosslinking stimulatory effects on NK cells (for anti CD38 moAb), direct cytotoxicity	Decrease target expression, complement protein increase, sBCMA increase, BCMA down expression	Change drug class, upregulation of CD38 using ATRA	[61-67]
IMIDs	Tha Lena Poma	BM microenvironment targeting; degradation of *IKZF1 *and *IKZF3* via *CRBN*-dependent ubiquitination; IRF4 and *MYC* downregulation; triggering caspase 8/9-mediated apoptosis; immune modulation; anti-angiogenic activity	Downregulation of *CRBN* expression; deregulation of *IRF4* expression Mutation of *CRBN* and *IKZF1* and *IKZF3*	Change drug class, next-generation *CRBN* E3 ligase modulators (iberdomide)	[58,59]
PIs	Bort Car Ixa	Inhibition of activity of the 20S proteasome; inhibition of NF-κB activity; induction of apoptosis by activation of caspase 8/9 and p53; adhesion molecules downregulation	Mutation of β5 subunit, upregulation of pumps of efflux, HSP induction	Change drug class, pan proteasome inhibitor (marizomib), hydroxychloroquine, pan HDAC inhibitor	[46,48]

C: Class of drug; D: drug name; M of A: mechanisms of action; DR: drug resistance; moAb: monoclonal antibody; IMIDs: immunomodulatory drug; PI: proteasome inhibitors; Dara: daratumumab; Isa: isatuximab; Elo: elotuzumab; Bela: belantamab mafodotin; Tha: thalidomide; Lena: lenalidomide; Poma: pomalidomide; Bor: bortezomib; Car: carfilzomib; Ixa: ixazomib; ADCC: antibody-dependent cellular cytotoxicity; CDC: complement-dependent cytotoxicity; BCMA: b cell maturation antigen; ATRA: all trans retinoic acid; *IKZF1*: Ikaros; *IKZF3*: Aiolos; BM: bone marrow; *CRBN*: Cereblon; IRF4: interferon regulatory factor 4; NF-κB: nuclear factor-κB; HDAC: histone deacetylase; HSP: heat shock protein.

### Drug resistance to proteasome inhibitors

The PIs used in recent years in clinical practice are bortezomib (Bor), carfilzomib (Car), and ixazomib (Ixa)^[39,40]^. Bor is approved for use at diagnosis in both transplant and non-transplant eligible MM patients, while Car and Ixa are used in the relapsed and refractory treatment setting. The structure of the proteasome was first described as a hollow single-cylinder protein. The proteasome functions as an ATP-dependent organelle in which almost 90% of intracellular proteins are degraded following tagging by the polyubiquitin chain. Three subunits of the proteasome are recognized as the site of degradation in the 20S particle: β1 (caspase), β2 (trypsin), and β5 (chymotrypsin). The final result of proteasome inhibition is MM plasma cell death or apoptosis via protein accumulation^[41]^. Other mechanisms have been reported: p53 activation, inhibition of nuclear factor-κB (NF-κB) activity, activation of c-Jun N-terminal kinase, and stabilization of cell cycle inhibitors. PI can bind the β5 subunit reversibly (Bor and Ixa) or irreversibly (Car). Resistance to PIs is related to mutations in the β5 subunit gene (*PSMB5*) at both diagnosis and relapse^[42,43]^. The presence of mutations at other subunits, namely *PSMA1*, *PSMB8*, and *PSMB9*, has been described, but they have not been found to be related to resistance yet and thus need to be confirmed. Another protein important for DR seems to be the X-box protein 1 that acts in the proteostasis in the MM plasma cell. Downregulation of this pathway has been reported in PI-DR cells^[44]^. Decreased drug accumulation is another mechanism of DR in MM. In particular, the multidrug resistance protein P-glycoprotein (P-gp) has been well studied in the past^[45]^. P-gp works by implementing drug efflux from the MM plasma cell, thus reducing drug activity. Both Car and Bor have been reported to be P-gp substrates^[46-48]^. The mechanisms of resistance escape are reported in Table 1^[49,50]^. The results of preclinical studies indicate that inhibition of various heat shock proteins (HSPs), e.g., HSP90, can increase the efficacy of PIs^[51]^. HSPs are induced by the transcriptional blockade of protein degradation, which can contribute to drug resistance. The results from early phase I trials combining HSP90 inhibitors with PIs have identified safe doses for both drugs. In a preclinical study using bor-resistant MM cell lines, the blockade of IGF-1 downstream effectors re-sensitized cell lines to bortezomib. Furthermore, the IGF-1R inhibitor OSI-906 induced more apoptosis than Bor alone, both *in vitro* and *in vivo*^[52]^.

Moreover, investigators used a mouse MM model to study gene-expression signatures (GES) related to Bor resistance. GES related to resistance included nuclear factor (erythroid-derived 2)-like 2 (NFE2L2), highly expressed as part of an antioxidant-response pathway^[53]^. Thus, MM cells with elevated antioxidant capacity before treatment might be resistant to bortezomib.

### Drug resistance to immunomodulatory drugs

The IMIDs currently used in clinical practice are thalidomide, lenalidomide, and pomalidomide (Thal, Len, and Pom, respectively). All three agents were initially used in the relapsed and refractory setting, but Len and Thal are now also approved for use at diagnosis^[54]^. IMIDs have antiproliferative properties and direct pro-apoptotic effects. Other mechanisms exhibited are anti-angiogenic and immunomodulatory effects, by activating NK and T cells. The E3 ligase protein Cereblon (*CRBN*) is targeted by IMIDs, in particular in complex with CRL4, ultimately leading to degradation of the Aiolos and Ikaros proteins and *IRF4* reduction^[55-57]^. This mechanism of action is the principal target of DR, mainly because of occurring mutations or gene expression modifications in this complex. It has been demonstrated that *CRBN* mutations appear after a relapsed/refractory disease occurs, probably due to clonal selection after long IMID therapy^[58]^. In addition, IRF4 has been investigated as a potential downstream target for DR in Waldenstrom macroglobulinemia after therapy with Len and Pom^[59]^. Methylation of *CRBN* has also been recently reported^[60]^.

### Drug resistance to monoclonal antibodies

Monoclonal antibodies directed at antigens present on the surface of plasma cells have recently entered the therapeutic armamentarium against MM. The approved moAb in clinical practice are daratumumab, elotuzumab, isatuximab, and belantamab mafodotin. Thse drugs were all tested as monotherapy first, and have since been proven to be more efficacious when combined with other agents in triplet or quadruplet^[61-65]^.

Even though daratumumab, an IgG 1 anti-CD38 moAb, has been found to be very efficacious, resistance can be observed during treatment. Although CD38 mutations are not described as potential mechanism of DR, it has been reported that low CD38 expression could be a cause of initial resistance to therapy. Moreover, CD38 expression reduction during treatment has been described as a major mechanism and occurs via the action of sheddases^[66]^. Another occurring event observed is upregulation of CD55 and CD59 complement inhibitors^[67]^. For other moAb, the DR mechanism is not currently known, although it is logical to think that, as per daratumumab, the loss of the target antigen is likely a major mechanism of resistance^[68-72]^.

### Extrinsic mechanisms of drug resistance

**Figure 1:**
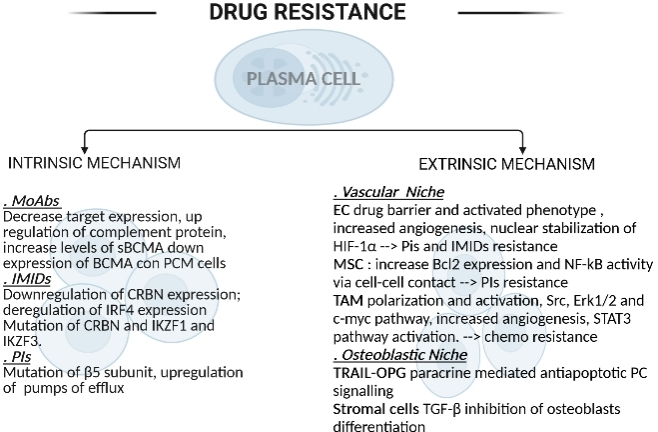
Drug resistance intrinsic and extrinsic mechanisms. EC: Endothelial cells; MSC: mesenchymal stem cells; TAM: tumor-associated macrophages.

The bone marrow microenvironment, including osteoblasts, osteoclasts, mesenchymal stem cells (MSC), and tumor-associated macrophages (TAM), is highly connected with MM progression and drug resistance [Figure 1]. In MM in the “osteoblastic niche”, macrophages are differentiated into osteoclasts by the release of osteoclasts activating factors (i.e., IL-6, IL-1-α, TNF-α, TNF-β, and IL-11) and promote bone resorption. Osteoprotegerin represents an antiapoptotic MM factor by binding to TNF-related apoptosis-inducing ligand. Stromal cells can give drug resistance by TGF-β inhibition of osteoblasts differentiation. Stromal cells in the microenvironment have been shown to secrete several cytokines that regulate the antiapoptotic members of the Bcl-2 family (Mcl-1, Bcl-xl, and Bcl2) via IL-6 signaling^[73]^. A “vascular niche” is formed by endothelial cells (EC), MSC, and TAM and can protect MM plasma cells from cytotoxic drugs. In particular, EC can express an aberrant active phenotype (VEGFR-2 and FGFR-3) that can help to prevent MM apoptosis, favoring PC migration into the bloodstream and dissemination^[74]^. MSC can contribute to bortezomib resistance in MM via Bcl2 increased expression and enhanced NF-κB activity through cell-cell contact^[74]^.

TAM have a basic role in MM pathogenesis, since they promote plasma cells proliferation, homing, and angiogenesis, supporting MM immune evasion and progression^[75]^.

MM plasma cells *in vitro* could upregulate CD206 expression and favor an M2 TAM polarization of cocultured macrophages^[76]^. In a preclinical model, CD68-positive TAM were shown to inhibit drug-induced apoptosis of tumor cells by caspase 3 and poly-ADP ribose polymerase cleavage^[77,78]^. Moreover, intracellular adhesion molecule-1 (ICAM-1) and P-selectin glycoprotein ligand-1 (PSGL-1) on the MM cells surface could activate TAM and favor TAM-induced chemoresistance through the *SRC*, *ERK1/2*, and *C-MYC* pathway^[79]^. VEGF production by M2 TAM was demonstrated during progression from MGUS to MM, with increased angiogenic switch^[80,81]^.

An interesting study reported a possible correlation between the pro-tumor effect of TAM and the Stat3 pathway activation in 5T33MM cells. Interestingly, an ATP-competitive Janus kinase (*JAK*)2 inhibitor, the so-called AZD1480, could restore the sensitivity of MM cells to bortezomib^[82,83]^.

Clinical studies confirmed CD68/CD163 double-positive M2 TAM were associated with increased microvessel density and reduced survival, independently of the MM stage^[84-86]^. In this field, high IHC CD163-positive M2 TAM expression at diagnosis was associated with lower complete response (CR) rate and reduced PFS and OS in 198 MM patients receiving bortezomib-based regimens^[84]^. Interestingly, an elevated level of soluble M2 TAM markers CD163 and CD206 was associated with worse OS; conversely, higher M1 density demonstrated a correlation with OS improvement^[85]^.

In a retrospective study enrolling 68 MM patients, an elevated CD68-positive and CD163-positive TAM expression showed a significant reduction of six-year OS, as confirmed by multivariate analysis. As a complementary finding, an increased CD163-positive M2 TAM number was associated with an elevated microvessel density^[86]^. In another relevant study, a reduced response was observed in patients presenting with high CD68-positive and CD163-positive TAM; however, only high CD163 TAM expression was related to a reduced PFS and OS. CD163 and CD168 were combined with ISS to design a new prognostic model^[87]^. M2 TAM infiltration and correlation with pro-angiogenic factor CD147 were investigated in a spectrum from MGUS to recurrence MM. CD163 was used as the M2 marker and the cutoff for M2 infiltration was 100 per core. The authors showed a significant OS reduction in relapsed MM patients with high M2 expression (32 months *vs*. 6 months, *P* = 0.02), suggesting a prognostic role of CD163-positive TAM in MM^[88]^. Finally, Andersen *et al*.^[89]^ evaluated CD163 as a soluble marker in 104 blood samples and 17 BM samples in newly diagnosed MM patients. CD163 BM expression was higher compared to blood samples and was associated with a higher ISS stage. An elevated CD163 expression, with the suggested cutoff of 1.8 mg/L, was associated with poor prognosis, further suggesting M2 TAM could favor MM growth and progression^[89]^.

## MINIMAL RESIDUAL DISEASE

New drugs in MM have revolutionized the treatment paradigm and improved both progression-free and overall survival. However, progress in the definition of response is needed, since a CR has been defined for almost 15 years simply as the absence of a monoclonal component at immunofixation and a percentage of monoclonal plasma cells < 5% in the bone marrow.

The goal is to predict patients relapsing soon after the initial therapy and distinguish them from patients who maintain a long response (sustained CR) that is a surrogate for PFS and OS. Novel drugs are combined now in triplet or quadruplet treatment schedules and can implement in a high percentage of patients CR to a virtual disease disappearance. However, most MM patients still relapse. NGF and NGS have been introduced into clinical trials, bringing more sensitivity to detect minimal residual disease after therapy^[23-34]^. The detection limit of MRD is now considered 10^-5^; however, data suggest that a deeper limit of 10^-6^ or 10^-7^ could predict a better PFS^[90]^. Although NGS has been reported initially to have a deeper limit of detection (10^-6^) than NGF (10^-5^), both can now reach comparable sensitivity at 10^-6^.

### NGF and NGS in MM

NGF has recently become part of the MRD evaluation, and it is based on the detection and quantification of normal PC *vs*. monoclonal PC using specific antibodies: PC markers such as CD38 and CD138; aberrant antigens expression such as CD45^-low^, CD19^-^, CD27^-^ , CD81^-^, and CD56; CD28; CD117; and light chains κ or ʎ. The Euro-Flow Consortium has introduced a more sensitive standardized technique based on two eight-color tubes that permit detecting MRD with 100% applicability^[24]^ with a 4 h sample processing time. Sensitivity needs to be at least 10^-5^, but it has been reported as even better. A correct and adequate concentrate sample is needed to avoid hemodilution, and strategies to overcome CD38 monoclonal antibody interference have been described^[26]^.

NGS analyzes the clonal rearrangements of the immunoglobulin heavy chain (IgH) regions with parallel sequencing of reads with a sensitivity of 10^-6^. Importantly, patient primer construction is not needed, and the depth of detection is a strength^[91]^. Commercial kits are now available and FDA approved (LymphoSIGHT® and ImmunoSEQ, Adaptive Biotechnologies). Costs and a bioinformatic database for analysis as well as hemodilution are the limits^[26]^. NGF and NGS are recognized as complementary techniques, and the IMWG suggests utilizing the one available at the centers in clinical trials^[36]^. The Cassiopeia trial showed comparable sensitivity for NGF and NGS^[13]^.

### Minimal residual disease sub-clone characterization

MRD can be a surrogate for PFS and OS and is becoming an important tool for risk-stratification since the depth of response is correlated with prolonged survival^[92]^. In fact, a non-sustained CR can frequently be seen in MRD-positive MM patients. A goal in actual MM therapy could be to reach a sustained CR with MRD negativity lasting for at least one year. Searching for a sub-clone in MRD analysis could be an ideal way to identify drug resistance. It has been reported that MRD MM sub-clones could overexpress CD11a, CD11c, CD29, CD44, CD49d CD49e, CD54, CD138, CXCR4, and HLADR in the GEM2010MAS65 study involving 40 elderly patients^[93]^ treated with nine cycles of VMP (bortezomib, melphalan, and prednisone) or alternating VMP to Rd (lenalidomide and dexamethasone). In particular, integrins, chemokines, and adhesion molecules were overexpressed. These chemoresistant clones with a specific multi-flow signature also displayed genetic copy number alterations (CNA) that were present since diagnosis, and the resistant clone was selected after therapy resistance. The same group recently studied a larger number of patients (*n* = 390) in the PETHEMA/GEM2012MENOS65 protocol (six induction cycles with Bor, Lena, and dexamethasone followed by autologous stem cell transplant, two consolidation cycles with Bor, Lena, and dexamethasone, and then randomization to maintenance therapy with Lena and dexamethasone *vs*. Ixa, Lena, and dexamethasone)^[88]^. They used NGF to identify detectable MRD and mechanisms related to resistance in 90 patients with high-risk (HR; i.e., del17p, t4;14, or t14;16) cytogenetics and 300 patients with standard-risk cytogenetics (SR; i.e., other anomalies not HR). Importantly, the results show the superiority of 90% PFS in MRD-negative patients *vs*. MRD-positive patients, irrespective of the cytogenetic status. Moreover, NGF studies and whole-genome sequencing showed clonal selection and higher genomic aberrations and mutations in 40 patients with multi-resistant clones^[94]^. Most mutations affected *KRAS*, *BRAF*, *CCND1*, *ROS1*, *NRAS*, and *FLT3* genes. CNA and mutations present at diagnosis were more likely to disappear in SR-cytogenetics patients, while HR patients had novel mutations during treatment, suggesting more genomic instability. In addition, when evaluating transcriptional clones, patients with HR cytogenetics showed the expression of PRDX6 and SOD1, which were related to a worst PFS. The authors concluded that, in HR cytogenetics MM patients, MRD resistance can arise from clones that evolve transcriptionally after therapy.

### Minimal residual disease negativity after current upfront therapies for transplant and non-transplant eligible myeloma patients

With the introduction of novel drugs, responses can be seen in the majority of MM patients treated at diagnosis, both transplant and non-transplant eligible, with PFS lasting up to five years [Tables 2 and 3].

**Table 2:** CR and MRD results in transplant eligible patients with currently available therapy

**Authors**	**Regimen**	***n***	**ASCT** **(NO/1/2)**	**C/M**	**CR (%)**	**MRD neg (%)**	**PFS/OS (months)**
Cavo *et al*.^[95]^	TD VTD	238 236	2	C + M	41 58	NA	40.7/110 59.6/NR
Attal *et al.*^[98]^	VRD VRD	350 350	NO 1	C + M	49 59	65 80	37/NR 50/NR
Jasielec *et al*.^[99]^	KRD	76	1	C + M	78.9	70	NR
Moreau *et al*.^[13]^	Dara-VTD VTD	543 542	1	C + M	39 26	64 44	NR
Voorhees *et al*.^[100]^ Laubach *et al*.^[101]^	Dara-VRD VRD	103 104	1	C + M	66 47	64 30	NR

PFS: Progression-free survival; OS: overall survival; CR: complete response; C + M: consolidation + maintenance; NA: not applicable; NR: not reached; ASCT: autologous stem cell transplantation; TD: thalidomide and dexamethasone; VTD: bortezomib, thalidomide, and dexamethasone; VRD: bortezomib, lenalidomide, and dexamethasone; KRD: carfilzomib, lenalidomide, and dexamethasone; Dara-VTD: daratumumab, bortezomib, thalidomide, and dexamethasone; Dara-VRD: daratumumab, bortezomib, lenalidomide, and dexamethasone.

**Table 3:** CR and MRD results in non-transplant eligible patients with currently available therapy

**Authors**	**Regimen**	***n***	**CR (%)**	**MRD neg (%)**	**PFS/OS (months)**
San Miguel *et al*.^[102]^	VMP MP	344 338	30 4	NA	24/56 16.6/43
Benboubker *et al*.^[103]^	RD cont. RD18 MPT	535 541547	15 14 9	NA	25.5/59 20.7/62 21.2/49
Facon *et al*.^[12]^	Dara-RD RD	368 369	48 25	31 10	NR 34/NR
Mateos *et al*.^[11]^	Dara-VMP VMP	356 350	46 25	28 7	36.4/NR 19.3/NR
Durie *et al*.^[105]^	VRD RD	235 225	24.2 12.1	NA	41/NR 29/69

PFS: Progression-free survival; OS: overall survival; CR: complete response.

It is now evident that PFS alone cannot be the main parameter to judge new drugs in clinical trials. MRD has been proven to be a good prognosticator for PFS, OS, and drug resistance^[92]^. In the GIMEMA-MMY-3006 study, VTD was compared to TD in 480 MM transplant eligible patients for induction therapy and double ASCT followed by consolidation therapy^[95]^. VTD incorporated into a double ASCT strategy plus consolidation therapy proved to be superior to TD in terms of CR attained (58% *vs*. 41%, respectively) and PFS and OS at 10 years (34% *vs*. 17% and 60% *vs*. 46%, respectively)^[96]^. Although MRD was not done at that time, one study showed that, in a subset of patients treated with VTD consolidation after ASCT, MRD negativity was achieved and relapses were delayed^[97]^. In the IFM 2009, VRD was compared with VRD + ASCT, and in the latter group CR was superior (59% *vs*. 49%) but OS did not differ significantly^[98]^. Importantly, MRD negativity was significantly correlated with improved three-year PFS (87% *vs*. 42%), independently of the therapy received. Car was combined with Len and dexamethasone in the KRD regimen tested as induction and consolidation + maintenance therapy for up to 10 cycles^[93,99]^. Although relatively few patients were treated, MRD negativity was reached in a high percentage of patients. Recently, Dara was added to VTD and compared to VTD alone in 1085 patients in the CASSIOPEIA study^[13]^: response and MRD negativity were superior in Dara-VTD *vs*. VTD (64 % *vs*. 44%). Dara was added before ASCT to VRD in the GRIFFIN study and then compared to VRD alone^[100,101]^. Consolidation and maintenance therapy were also applied for up to 26 months of total therapy. MRD negativity was significantly superior in the Dara group (64% *vs*. 30%).

In the non-transplant eligible setting, great improvement with respect to the standard VMP or RD used until a few months ago^[102-104]^ was brought recently by the addition of daratumumab. The MAIA study^[12]^ compared Dara-Rd *vs*. Rd in MM patients not eligible for ASCT. At five years of follow-up, PFS has not been reached for the Dara-Rd group. CR was reached in 47% *vs*. 24% and MRD negativity was significantly higher (31% *vs*. 10%). The Alcyone trial^[11]^ compared VMP *vs*. Dara-VMP. This study confirmed the superiority of the triplet with Dara, in both PFS and MRD negativity. Lastly, VRD was superior to RD in the SWOGS0777 trial in patients not proceeding to ASCT (PFS 41 months *vs*. 29 months)^[105]^. Daratumumab was tested as consolidation therapy in patients achieving a > VGPR after ASCT in the DART4MM study^[106,107]^. An interim analysis showed MRD negativity in 45% of the patients at six months of treatment. Besides the great progress achieved with the new drugs with regard to the depth of responses, MRD status should not guide clinical decisions.

## CONCLUSION

MM patients still relapse after a variable period of remission. Drug resistance is a major cause of MM relapse with both intrinsic and extrinsic mechanisms. Therapeutic targeting of the bone marrow microenvironment and its interaction with MM plasma cells seems to be an ideal approach for the future. Besides the need for novel, more efficacious drugs, better prognosticators are also needed. MRD measured by NGF or NGS has entered into the MM diagnostic armamentarium and is a great prognosticator for novel drugs in clinical trials. Sustained MRD negativity is likely to be a long-term remission pre-requisite. Research has to be done to better clarify its role in identifying minimal residual sub-clones resistant to drugs commonly utilized in MM. NGF seems promising in this biological view. The characterization of these clones should be pursued in future large MM trials.

## DECLARATIONS

### Copyright

© The Author(s) 2022.
